# Data Gathering Bias: Trait Vulnerability to Psychotic Symptoms?

**DOI:** 10.1371/journal.pone.0132442

**Published:** 2015-07-06

**Authors:** Ana Catalan, Claudia J. P. Simons, Sonia Bustamante, Nora Olazabal, Eduardo Ruiz, Maider Gonzalez de Artaza, Alberto Penas, Claudio Maurottolo, Andrea González, Jim van Os, Miguel Angel Gonzalez-Torres

**Affiliations:** 1 Department of Neuroscience, University of the Basque Country, Basque Country, Spain; 2 Department of Psychiatry, Basurto University Hospital, Bilbao, Spain; 3 Department of Psychiatry and Psychology, South Limburg Mental Health Research and Teaching Network, EURON, Maastricht University Medical Centre, Maastricht, The Netherlands; 4 GGzE, Eindhoven, The Netherlands; 5 Clínica Servicios Médicos AMSA, Bilbao, Vizcaya, Spain; 6 King’s College London, King’s Health Partners, Department of Psychosis Studies, Institute of Psychiatry, London, United Kingdom; University of Hertfordshire, UNITED KINGDOM

## Abstract

**Background:**

Jumping to conclusions (JTC) is associated with psychotic disorder and psychotic symptoms. If JTC represents a trait, the rate should be (i) increased in people with elevated levels of psychosis proneness such as individuals diagnosed with borderline personality disorder (BPD), and (ii) show a degree of stability over time.

**Methods:**

The JTC rate was examined in 3 groups: patients with first episode psychosis (FEP), BPD patients and controls, using the Beads Task. PANSS, SIS-R and CAPE scales were used to assess positive psychotic symptoms. Four WAIS III subtests were used to assess IQ.

**Results:**

A total of 61 FEP, 26 BPD and 150 controls were evaluated. 29 FEP were revaluated after one year. 44% of FEP (OR = 8.4, 95% CI: 3.9–17.9) displayed a JTC reasoning bias versus 19% of BPD (OR = 2.5, 95% CI: 0.8–7.8) and 9% of controls. JTC was not associated with level of psychotic symptoms or specifically delusionality across the different groups. Differences between FEP and controls were independent of sex, educational level, cannabis use and IQ. After one year, 47.8% of FEP with JTC at baseline again displayed JTC.

**Conclusions:**

JTC in part reflects trait vulnerability to develop disorders with expression of psychotic symptoms.

## Introduction

Patients with a diagnosis of psychotic disorder show a tendency to make decisions on the basis of little or no supporting evidence [1]—they ‘‘jump to conclusions” [2]. Patients thus show a tendency to seek less information to reach a decision, which has been referred to as a data-gathering bias [3–5].

Whereas some studies have found jumping to conclusions (JTC) to be associated with a diagnosis of schizophrenia [6–10], others suggest it is specifically associated with delusions [5,11–17] or even delusion-proneness [18–21]. JTC is also present in first episode psychosis (FEP) [22–25].

Recent studies have not been able to detect substantial associations between JTC and paranoid or delusional symptomatology [23,26–30] and some studies did not find differences in the prevalence of JTC in patients versus controls [31]. On balance, although an association with psychosis is likely, the literature on JTC does not permit a definitive conclusion as to whether it relates more to psychotic symptoms, such as delusions, or diagnostic categories associated with trait psychosis proneness [1]. As a trait, it could contribute to the formation of delusions and be part of the vulnerability of psychotic disorder; as a state, it could be a factor mediating the maintenance of transdiagnostic delusionality [1]. In order to further examine the trait-or-state issue it may be productive to examine JTC in relation to low grade, or subclinical, psychotic experiences in other diagnostic categories. For example, anorexia patients did not display elevated JTC and JTC was not associated with level of delusionality in this group [32]. Similarly, patients with body dysmorphic disorder (BDD) did not display elevated JTC relative to controls although poor insight in BDD was associated with elevated JTC [33]. Patients with obsessive-compulsive disorder did not display elevated JTC, howevwr, JTC was associated with delusionality in this group [34].

Personality disorders per definition reflect trait alterations in mental function. Borderline personality disorder (BPD) is associated with trait elevation of psychotic symptoms [35] and according to one previous study may be associated with JTC [36]. About 20–50% of patients with BPD experience psychotic symptoms (hallucinations and paranoid ideation) [35]. Hallucinations can be similar to those in patients with psychotic disorders in terms of phenomenology, emotional impact and their persistence over time [35]. BPD patients therefore may represent an important population for understanding cognitive processes associated with delusion formation. We therefore wished to study JTC reasoning bias in relation to psychosis in BPD patients.

The mechanisms underlying JTC remain unclear. It has been argued that higher order cognitive processes such as threat-related confirmatory reasoning [37], an intolerance to ambiguity [38], overestimation of the conviction in choices at the beginning of the decision-making process [2,39,40], a lowered threshold for making decisions [41–44], a ‘‘need for closure” [45], or alterations in working memory [46] may account for the development of a rushed reasoning style.

Emerging literature suggests that persons with delusions are over-confident in their incorrect decisions [44,47–54]. Some of these biases have been found to correlate with positive symptoms (i.e., delusions and hallucinations), which some consider the core of schizophrenia and related disorders. It is possible that, in addition to a cognitive reasoning bias, other factors, such as impulse control, mediate the association between JTC and positive symptoms. However, no associations between impulse control and delusions or between impulse control and JTC have been reported [9].

The contribution of IQ to JTC has been partially studied [16]. This is surprising given that reasoning ability is a central subconstruct within the structure of IQ. Where IQ scores have been covaried, results have been inconsistent. While some studies have reported evidence that JTC was associated with lower IQ scores [5,12], others have not [55]. The relation with working memory has been more analyzed, nonetheless data are inconclusive [9, 46].

Given the fact that BPD is associated with trait psychosis proneness, investigating this group in a comparison with patients with psychotic disorder and well controls may be productive. If JTC represents a trait, it should be associated with BPD and FEP but not necessarily with level of psychotic symptoms within BPD and FEP. Furthermore, it would show a degree of stability over time in FEP. If it represents a state, it may not be associated with BPD, but may be associated with delusionality across the different groups.

The aims of this study were to (i) determine the rate of JTC in a group of patients with FEP, BPD and controls, (ii) asses the relationship between JTC and psychosis liability, (iii) measure the stability of JTC in FEP over the course of one year, (iv) investigate associations between JTC and positive psychotic symptoms across different groups, and (v) assess associations between JTC and IQ.

## Materials and Methods

### Ethics statement

The local ethics committee (Ethics Committee of Clinical Research of Basurto University Hospital) approved the study design and the patients provided written informed consent. We obtained written consent from all patients of the study. In case of minors, written informed consent was sought from both the participant and their guardians on their behalf.

The study was observational and in part prospective, in three groups of subjects: FEP patients, BPD patients and well controls. The study was carried out in Basurto University Hospital (HUB–Bilbao—Spain). The key outcome was the “number of draws to decision” in the neutral beads task.

### Sample

Data were collected in a convenience sample of patients with a diagnosis of FEP, admitted consecutively to the inpatient unit of HUB from January 2009 to September 2013. BPD patients were recruited at the Day Hospital of HUB and AMSA Clinic. Controls were recruited from the general population in the same catchment area as the patients, through advertisements and announcements. Controls did not report first-degree relatives with a psychotic disorder. Patients were examined when the psychiatrist in charge considered that they were stable and were able to provide informed consent. Inclusion criteria were the following (for the three groups): between 17 and 65 years of age, sufficient mastery of the Spanish language, IQ >70; for FEP patients: exposure to antipsychotic medication < 1 year. The psychotic episode fulfilled DSM-IV-TR criteria for affective or non-affective psychotic disorder; for BPD patients: meeting DSM-IV-TR criteria for BPD, in the absence of psychotic disorder comorbidity. Exclusion criteria for FEP patients were: psychotic episode was the consequence of abuse of drugs or somatic disorder and for all three groups: unwillingness to participate.

Sociodemographic variables were collected including age, sex, onset of illness (defined as age at first treatment), duration of untreated psychosis, employment status, marital status and living arrangement (alone, with parents, with partner/family). In the patient group, clinical scales such as the PANSS (Positive and Negative Syndrome Scale) [56] (positive, negative, general and global domains) and GAF (Global Assessment of Functioning) [57] were used to assess (functional impact of) psychopathology. The Operational Criteria Checklist for Psychosis [58] was completed, based on clinical instruments and relevant data in the medical history, and used to establish the diagnosis of the patients using the associated OPCRIT computer programme [59].

### Instruments

Beads task: The probabilistic reasoning task [60] was used to establish the rate of JTC response patterns. Participants were shown two jars of coloured beads in equal but opposite ratios (60 red: 40 blue; 60 blue: 40 red). Participants were told that one jar had been chosen and that beads would be drawn from this jar and shown to the participant. The participant had to decide from which jar the beads were coming. Beads task materials were presented on a laptop computer.

IQ: The short form of the Wechsler Adult Intelligence Scale (WAIS)–III [61] was assessed for an indication of intellectual functioning (IQ), and included the following tests: ‘Block Design’, ‘Digit Symbol’, ‘Arithmetic’ and ‘Information’.

SIS-R: Structured Interview for Schizotypy—Revised [62]. The Structured Interview for Schizotypy—Revised was used to determine a broad range of schizotypal symptoms and signs. Items can be scored on a 4-point scale from absent (0) to severe (3). Positive schizotypy covers the symptoms referential thinking (2 items), magical ideation, illusions, psychotic symptoms, and suspiciousness (6 items). Negative schizotypy covers the symptoms of social isolation, introversion, restricted affect, and poverty of speech (4 items). Mean schizotypy scores for these dimensions were calculated, resulting in a positive schizotypy and a negative schizotypy score. This scale was used in the control group.

CAPE: The Community Assessment of Psychic Experiences [63] was used to assess the lifetime prevalence of positive, negative and depressive symptoms. This self-report scale measures positive, negative and depressive symptoms along a frequency scale (0 = never to 4 = nearly always) and a distress scale (1 = not distressed to 4 = very distressed). The scale was used in the control and BPD groups. The mean CAPE positive, negative, and depressive score was the mean of the positive, negative, and depressive symptom frequency scale, respectively.

PANSS: This scale contains 7 positive symptom subscale symptoms, 7 negative symptom subscale symptoms and 16 general psychopathology symptoms. Each item can be rated from 1 (absent) to 7 (extreme).

### Analyses

A Kolmogorov-Smirnov test was used to test for deviation from normality. ANOVA was used to examine differences in continuous variables, and Kruskal-Wallis test was used for non-normally distributed variables. In the case of categorical variables, chi-square tests, and Fisher´s exact test, when indicated, were performed.

#### Association between JTC and psychosis liability

A 3-level group variable was constructed reflecting the hypothesized order in liability for psychosis, with controls (coded 0), BPD (coded 1), and FEP (coded 2) in the highest category. The number of beads requested by a subject yielded a continuous outcome variable within a range from 0 to 20. This variable was found to be non-normally distributed. In order to examine the JTC outcome, a variable was constructed indicating whether a subject showed a JTC reasoning bias, defined as requesting only 1 bead before deciding (JTC1), conforming to previous work [5]. This cut-off was chosen as it reflects the most definite expression of the reasoning bias under investigation and should therefore be more discriminating between groups. In order to test this assumption, associations were also tested using less stringent cut-off values (i.e., using 2, 3, or more beads before deciding).

The association between JTC1 and psychosis liability (the 3-level group variable reflecting liability for psychosis) was examined using logistic regression analysis, and progressively less stringent selections of JTC cut-offs were also considered. Effect sizes were expressed as odds ratios with their 95% confidence intervals. The following a priori selected confounders of the association between JTC1 and psychosis liability were included in the logistic regression model: age, sex, general intelligence, level of education (years of education) and current use of cannabis.

#### JTC stability in FEP

A McNemar´s test was performed to assess the stability of JTC1 in the FEP group at one year follow-up.

#### Association between JTC and positive psychotic symptoms

We divided the PANSS positive symptom score by its tertiles, creating 3 tertile groups in the FEP group. The SIS-R and CAPE scale were used in the control group, and the CAPE in the BPD group, and similarly analysed as tertile groups.

Associations between JTC1 (dependent variable) and presence of positive symptoms as measured with the PANSS, CAPE and SIS-R (independent variable) were examined using logistic regression analysis.

In order to assess whether any association with delusions was independent from other positive and negative or depressive symptoms, subsequent analyses were performed in which all symptom domains, assessed with the PANSS, CAPE and SIS-R, were entered simultaneously in the model.

In order to assess the association between JTC1 and delusions and hallucinations in the FEP group, the score of each symptom on the PANSS positive symptoms scale was examined separately in the logistic model of JTC1.

#### JTC and IQ

The association between JTC1 and IQ was analysed using Mann Whitney.

Statistical analyses were carried out with STATA version 12 [64].

## Results and Discussion

### Results

#### Sample characteristics

61 FEP, 26 BPD and 150 controls were assessed at baseline. 29 FEP were re-evaluated after one year. FEP, BPD and control subjects did not show statistically significant differences in age and sex while differing in years of education (p<0.0001), marital status (p<0.01), socioeconomic status (p<0.01), living arrangement (p<0.01), work status (p<0.001) and IQ (p<0.0001) (Table 1). CAPE data were missing for two BPD patients. Diagnoses in the FEP group were: schizophrenia or schizophreniform disorder (n = 34), affective psychoses (n = 17), brief psychotic episode (n = 2) and delusional disorder (n = 8). All FEP patients were taking antipsychotic medication at the time of the assessment. Clinical variables are summarized in Table 2.

**Table 1:** Sociodemographic variables.

		*FEP (n = 61)*	*BPD (n = 26)*	*Controls (n = 150)*
		*N (%) Mean (SD)*	*N (%) Mean (SD)*	*N (%) Mean (SD)*
Sex	Male	39(64%)	11(42.3%)	87(58%)
	Female	22(36%)	15(57.7%)	63(42%)
Age (years)		35.5(13.0)	35.2(10.3)	33(11.3)
Years of education		15.1 (3.3)	17 (2.2)	17.8 (2.4)
Socio-economic level	High middle class	8 (13.1%)	6 (24%)	28 (18.7%)
	Middle class	34 (55.7%)	15 (60%)	109 (72.7%)
	Low middle class	19 (31.2%)	4 (55.7%)	13 (8.7%)
Marital status	Single	38 (62.3%)	15 (60%)	82 (54.7%)
	Married/Partner	16 (26.2%)	7 (28%)	65 (43.3%)
	Divorced	5 (8.2%)	3 (12%)	3 (2%)
	Widower	2 (3.3%)	0	0
Work status	Inactive	28 (45.9%)	19 (76%)	20 (13.3%)
	Active	28 (45.9%)	2 (8%)	85 (56.7%)
	Student	3 (4.9%)	1 (4%)	40 (26.7%)
	Retired	2 (3.3%)	0	2 (1.3%)
	Others	0	3 (12%)	3 (2%)
Living arrangement	Parents	32 (52.5%)	12 (48%)	64 (42.7%)
	Partner/Family	19 (31.1%)	7 (28%)	76 (50.7%)
	Alone	10 (16.4%)	6 (24%)	10 (6.7%)
WAIS-IQ		93.1 (16.4)	93.5 (15.0)	110.3 (14.5)

**Table 2:** Clinical variables.

	*FEP (N = 61) Mean (SD)*	*FEP FU (n = 29) Mean (SD)*	*Controls (n = 150) Mean (SD)*	*BPD (N = 26) Mean (SD)*
*PANSS positive*	29.3 (9.7)	9.2 (5.0)		
*PANSS negative*	10.7 (6.9)	11.7 (8.0)		
*PANSS general*	39.5 (11.4)	23.9 (9.9)		
*PANSS total*	79.2 (19.0)	44.0 (19.7)		
*SIS-R positive*			1.5 (1.6)	
*SIS-R Negative*			1.6 (1.3)	
*CAPE positive*			4.0 (2.7)	12.3 (8.3)
*CAPE negative*			6.7 (4.2)	16.0 (9.0)
*CAPE depressive*			4.8 (2.5)	12.6 (6.6)

FEP FU: first episode psychosis at one year follow-up. 44% of FEP displayed jumping to conclusions (one draw before taking a decision) vs 9% of controls and 19% of BPD patients (p<0.0001).

#### Association between JTC and psychosis liability

A linear trend was apparent in the association between JTC1 and psychosis liability, the rate of JTC being higher as the level of psychosis liability increased (OR linear trend = 2.9, 95% CI: 2.0–4.2). When entered as 2 dummy variables comparing associations with the reference control group, the difference in odds ratio reached significance for the FEP group (OR = 8.4, 95% CI: 3.9–17.9). In the BPD group, the OR was directionally positive but statistically imprecise (OR = 2.5, 95% CI: 0.8–7.8). The strength and statistical precision of the association between JTC1 and the psychosis liability variable was reduced when adjusted for age, sex, years of education, IQ, and use of cannabis (FEP: OR = 5.2, 95% CI: 2.1–13.0; BPD: OR = 1.5, 95% CI: 0.4–5.2) (Table 3). This reduction was partially attributable to IQ. As the cut-off criterion of number of beads used to define JTC became progressively less stringent, the association between JTC and schizophrenia liability was progressively weaker.

**Table 3:** Association between jumping to conclusions (beads<1) and the group variable reflecting psychosis liability (linear trend and as 2 dummy variables compared with control group).

*JTC*	*Odds ratio*	*p*	*95% CI*
PL (linear trend; unadjusted)	2.9	<0.0001	2.0–4.2
PL (linear trend)^a^	2.7	<0.0001	1.7–4.2
PL (linear trend)^b^	2.5	<0.0001	1.6–3.8
PL (linear trend)^c^	2.3	<0.0001	1.4–3.6
BPD vs controls	2.5	0.1	0.8–7.8
FEP vs controls	8.4	<0.0001	3.9–17.9
BPD vs controls ^a^	2.3	0.2	0.7–7.3
FEP vs controls ^a^	7.3	<0.0001	3.1–17.4
BPD vs controls ^b^	1.8	0.3	0.5–5.9
FEP vs controls ^b^	6.0	<0.0001	2.6–13.9
BPD vs controls ^c^	1.5	0.5	0.4–5.2
FEP vs controls ^c^	5.2	<0.0001	2.0–13.0

PL = psychosis liability, Group = 0: controls; group = 1: BPD; group = 2: FEP

^a^ adjusted for age, gender, years of education and use of cannabis

^b^ adjusted for general intelligence

^c^ adjusted for age, gender, years of education, use of cannabis and general intelligence

After one year, of the 27 FEP patients who displayed jumping to conclusions at baseline, 11 (47.8%) still displayed jumping to conclusions at follow-up whereas 12 did not display jumping to conclusions (p<0.001); 4 patients were not re-evaluated.

#### Association between JTC and positive psychotic symptoms

In the FEP group, there was no significant association between JTC1 and positive symptoms on the PANSS scale (OR = 1.0, 95% CI: 0.9–1.0). The association did not change after adjustment for age, sex, years of education, IQ, and use of cannabis (OR = 1.0; 95% CI: 0.9–1.0).

When entered as 2 dummy variables for comparison with the reference category of no delusions the association did not change (OR = 0.8; 95% CI: 0.2–2.7) (OR = 1.0; 95% CI: 0.4–3.0).

Examining the association between each of the symptom domains of the PANSS positive scale with JTC1 did not yield positive associations (Table 4). Similarly, there was no association between JTC1 and the CAPE positive symptom scale in the BPD group (OR: 2.2; 95% CI: 0.2–24.2) or in the control group (OR: 0.3; 95% CI: 0.1–1.02). Results were similar for the SIS-R scale in the controls (OR: 0.9; 95% CI: 0.5–1.9).

**Table 4:** Association between JTC and positive symptoms in patients with FEP.

*JTC and positive symptoms on PANSS*	*Odds ratio^a^*	*p^a^*	*95%CI^a^*	*Odds ratio^b^*	*p^b^*	*95%CI^b^*
Delusions	1.0	0.9	0.7–1.4	0.9	0.8	0.5–1.6
Thinking disorganization	1.0	0.9	0.8–1.3	1.0	0.9	0.7–1.5
Hallucinations	1.0	0.4	0.9–1.3	1.2	0.2	0.9–1.7
Agitation	1.0	0.8	0.7–1.3	1.0	0.8	0.7–1.4
Grandiosity	0.8	0.09	0.7–1.0	0.8	0.04	0.6–1.5
Suspiciousness	1.0	0.6	0.8–1.4	1.0	0.5	0.8–1.5
Hostility	1.0	0.9	0.7–1.3	1.1	0.5	0.7–1.8

^a^ calculated with JTC and 1 symptom domain at the time.

^b^ calculated with JTC and all symptom domains entered simultaneously.

#### Association between IQ and JTC

There was a significant association between JTC1 and IQ. Those with evidence of jumping to conclusions showed a lower IQ than non-jumpers (respectively mean = 94.8; SD = 19.2 vs mean = 106.2; SD = 15.9, p<0.001). However, differences as a function of JTC1 were only significant in BPD group (mean = 85; SD = 26.7 vs mean 95.5; SD = 11, p<0.01), although directionally similar in the FEP group (mean = 90.3; SD = 17 vs mean = 95.3; SD 17, p = 0.6) and the control group (mean = 107; SD = 14.8 vs mean = 110; SD = 14.5, p = 0.9).

Nevertheless, despite the IQ, the difference in JTC between FEP and controls remains statistically significant (Table 3). In the BPD group the difference with controls is not statistically significant. Furthermore if difference in JTC between BPD and FEP (no differences in IQ) are analysed it is still significant (OR: 3.4, 95%CI: 1.1–10.4, p< 0.05) suggesting IQ is involved in such differences.

### Discussion

Whether JTC has the quality of a state or a trait remains unclear. If it represents a state, it would be a dynamic characteristic that can change over time. In the case of a trait, it would be a stable characteristic, independent of fluctuation in factors such as delusions or hallucinations. As a trait it could contribute to the formation of delusions and be part of the vulnerability underlying psychotic disorder; as a state it could be a factor mediating the maintenance of delusions [5,7,15]. Studying people with subclinical psychotic experiences as part of a stable personality trait, such as patients with BPD, may shed further light on this issue. As far as we are aware, this is the first study to compare JTC style in FEP, BPD and controls.

Our results demonstrate that a JTC reasoning bias is associated with psychotic disorder but not with the level of psychotic psychopathology or individual symptoms. Furthermore, the longitudinal data, in agreement with other work [26], demonstrate that JTC shows a degree of stability over time.

BPD patients, as a group with trait delusion-proneness are a useful population for understanding cognitive processes that are present before delusion formation. Only one previous study examined JTC in BPD in comparison to controls, but not FEP [6]. In the current study, the small number of patients in the BPD group resulted in low power–the OR, however, suggests association (OR = 2.5; n = 26). Indeed, when tested as a linear trend indexing transdiagnostic psychosis liability from controls to BPD to FEP, the association was large and significant.

In the current study, the rate of JTC was similar to that reported in previous studies of groups with longer histories of psychotic illness [8]. There is little in the literature on the performance of people with FEP. FEP patients may represent an especially important population for exploring and testing hypotheses regarding JTC bias, because they are less likely to be affected by the potential consequences of psychotic illness over time. Furthermore, they will not have had years of exposure to antipsychotic medications, which may confound outcome measures of cognitive function [65,66]. This is more difficult to control in BPD as patients are often prescribed long term medication for several problems. In this study, medication use by BPD patients was not accounted for.

More than 40% of FEP patients with JTC reasoning bias showed continuity of JTC bias over the course of one year, lower than a recent study which showed an 8-month stability rate of more than 70% [67]. Consistent with this, Moritz and Woodward [8] found that JTC did not normalize even after delusions had abated.

It is generally considered that cognitive impairment in psychosis is a trait-like phenomenon [68]. Thus, JTC may relate to a data-driven processing style, as a tendency towards limited data gathering. The role of general intelligence and cognitive abilities in reasoning bias remains under-researched. In previous studies, no evidence was found for a role of memory impairment [11], and mixed evidence for mediation was found for the ability to process sequential information [15,21].

However, some studies have described a significant association between JTC and general intelligence [5]. In the current study, FEP and BPD displayed lower IQ than controls. Part of the association between JTC and these mental disorders may thus be mediated by cognitive alterations associated with psychosis. The current study found an association between IQ and JTC in a sample of FEP and BPD, and part of the association between JTC and FEP and BPD was reducible to IQ. Nevertheless, the association between JTC and FEP remained large and significant, as did the association between JTC and linear psychosis proneness. The difference between the BPD group and controls is not statistically significant possibly due to the small number of subjects in this group.

Within the three groups, the level of positive symptoms was not associated with JTC reasoning bias, suggesting that JTC reflects a more general vulnerability to disorders with psychosis proneness rather than specific symptoms in general. However, the state-trait dichotomy in itself is somewhat problematic, as is the inference that the difference between trait and state determines whether a certain mechanism is involved in the formation or in the maintenance of delusions. First, a factor with a trait quality can be a necessary but not yet sufficient condition to develop a symptom, and a covarying state quality does not add much information with regard to etiological mechanisms. There may be other factors that make the trait come to expression, just like a (genetic) predisposition can come to expression under certain conditions [69,70]. As Bentall [71] stated, the assumption behind the dichotomous trait-state distinction is that abnormalities are either present prior to the emergence of symptoms (in which case they may play a causal role) or covary with symptoms (in which case they may be either part of the symptom picture or epiphenomena). Thus, this is not the only possible relationship between JTC and symptoms. It may be that cognitive performance underpinning JTC in psychotic patients can be normal under optimal environmental circumstances and become pathological if these circumstances become unfavourable.

Proponents of the continuity model of psychosis argue that psychosis should not be regarded as something that is either “present” or “absent” but may rather be represented as a continuum [72,73]. Garety and Freeman [16] argued that individuals with schizophrenia have a data-gathering bias, which may lead to their making hasty decisions that ultimately influence the formation of delusional beliefs. While this theory is plausible, most research to date has tested only those who already have delusions, which is problematic since it does not eliminate the possibility that individuals simply demonstrate such reasoning biases after they become delusional. The current study, in conjunction with previous studies investigating those who are delusion-prone [21], suggests that those who are more delusion-prone (BPD patients) process information in a similar way as those with active delusions: they jump to conclusions.

A limitation of this study is that the FEP and BPD groups can be too heterogeneous limiting the validity of any general comparison. As this is a convenience sample we did not have a sufficient number of subjects to perform a desirable analysis of different subgroups of symptomatology in FEP (schizophrenia, schizoaffective, mania…) and BPD (impulsivity, interpersonal sensibility…) to determine if they have a common underlying mechanism of JTC."Besides, assumption of a dimension of similar transdiagnostic psychosis liability does not make allowances for the possibility that our methods of assessing delusions in research fail to capture important differences between superficially similar but perhaps different psychopathology in different disorders.

## Conclusions

It seems unlikely that a single factor such as a JTC reasoning bias can be causally linked with either the formation or the maintenance of delusional beliefs. More likely, a dynamic interplay exists between delusional symptoms and cognitive processes. For example, it is possible that cognitive alterations in patients with FEP and BPD are not dysfunctional under optimum environmental conditions but, because of their reciprocal influences, are more easily “disturbed” by adverse events than those of individuals who have never had psychotic experiences [71,74]. This study is further evidence that rather than being a correlate of frank psychosis, the tendency to JTC may vary continuously across categories of mental illness. Data gathering bias may distinguish people with vulnerability to develop psychotic symptoms in a continuum from health to illness.
