# Correction: Data Gathering Bias: Trait Vulnerability to Psychotic Symptoms?

**DOI:** 10.1371/journal.pone.0135761

**Published:** 2015-08-07

**Authors:** Ana Catalan, Claudia J. P. Simons, Sonia Bustamante, Nora Olazabal, Eduardo Ruiz, Maider Gonzalez de Artaza, Alberto Penas, Claudio Maruottolo, Andrea González, Jim van Os, Miguel Angel Gonzalez-Torres

The eighth author’s name is spelled incorrectly. The correct name is: Claudio Maruottolo.
